# Supplementary material for: The role of cooking practices in the transmission of the foodborne parasite Taenia solium: A qualitative study in an endemic area of Southern Tanzania
Source: PLoS One. 2022 Oct 5;17(10):e0274877. doi: 10.1371/journal.pone.0274877 (PMC9534410; doi:10.1371/journal.pone.0274877)
Supplement: Attachment — Submitted filename: Reviewer comments.docx [file pone.0274877.s001.docx]

**Reviewer comments**

The paper provides important information on aspects which need to be well understood before we can achieve elimination of the parasite through change of practice. The paper is well written and should be considered for publication after addressing the comments raised.

Overall, the abstract there is no mentioned of the different communities of practice which seems to be the basis of this paper. I feel the authors should consider adding a sentence on this aspect in the abstract.

The discussion is unnecessarily long with some overlaps, the authors should consider making it more precise to shorten it. The authors should consider also comparing their finding with other studies particularly from Zambia (<https://pubmed.ncbi.nlm.nih.gov/27369573/>), Uganda (<https://www.sciencedirect.com/science/article/pii/S2211912418300968>) and (<https://www.frontiersin.org/articles/10.3389/fvets.2022.833721/full>)

**Specific Comments**

Line 73: It is an unclear what the authors mean by the term “Social situations”

Line 123 to 124: How did the researchers ensure that the women they spend time with did not change their practices because they knew they were being watched. How does the authors take care of this in their analysis and conclusions?

Line 134: The authors mention that the home cooks pass knowledge to female children. Why female children only?. In many African communities household chores including cooking is shared among the children without regard to their sex unless it was strictly not the case in the study community. In that case that needs to be clarified.

Line 145: The points in the figures e.g 35.954 amy be taken as decimal points but I believe they are commas to denote thousands?

Line 157 to 159: The naming of the villages may bring some confusion. One may think they have different practices due to their different geographical or physical context which seems to be implied by the names. The author should consider adding a statement to clarify this and make it clear from start.

Line 161: What defines a professional cook. This reference is misleading as it may mean cook who have undergo a formal training on cooking. A different term should be considered.

Line 337 to 338: The conclusion on that sentence may not be correct. There could be other factors which may make the cooks not adopt certain practices but not necessarily the inappropriateness of their knowledge. Consider revising.

Line 630: The professional cook as you call them may have also attained their knowledge from home just like the home cooks long before they ventured into the cooking business. This need to be clarified.

Line 739 to 743: The explanation given as to why the researchers relied on the DVO to select some of the villages is not sufficient. Most officers in Africa no the importance of avoid bias in selection of study participants or study sites. Explaining the need for randomization to the officers would have made them understand and allow the researchers to follow the study procedures in selecting the study sites. This form of bias would have been avoided.
